# Acceptability of levofloxacin dispersible and non-dispersible tablet formulations in children receiving TB preventive treatment

**DOI:** 10.5588/ijtldopen.23.0462

**Published:** 2024-02-01

**Authors:** D. T. Wademan, H. R. Draper, S. E. Purchase, M. Palmer, A. C. Hesseling, L. Van der Laan, A. J. Garcia-Prats

**Affiliations:** ^1^ Stellenbosch University, Desmond Tutu TB Centre, Department of Paediatrics and Child Health, Cape Town, South Africa;; ^2^ University of Wisconsin-Madison, School of Medicine and Public Health, Department of Pediatrics, Madison, WI, USA

**Keywords:** LTBI, paediatric TB, TB preventive treatment, latent TB infection, TPT

## Abstract

**BACKGROUND:**

We evaluated the palatability and acceptability of a 100 mg dispersible and a non-dispersible 250 mg levofloxacin (LVX) tablet formulation in children.

**METHODS:**

Perform was a randomised, open-label, cross-over trial of the relative bioavailability of LVX dispersible vs. crushed non-dispersible tablets in children aged <6 years routinely receiving TB preventive treatment. Children and caregivers completed Likert- and ranking-type measures on the acceptability of both formulations. We used summary, comparative and ranking statistics to characterise formulation acceptability.

**RESULTS:**

A total of 25 children were enrolled (median age: 2.6 years, IQR 1.6–4.0). Caregivers reported frequent challenges with preventive therapy in routine care prior to study entry, including taste of tablets (*n* = 14, 56%), vomiting/spitting out medicines (*n* = 11, 44%), and children refusing medicines (*n* = 10, 40%). Caregivers reported that the dispersible formulation was easier for their child to take than the non-dispersible formulation (*P* = 0.0253). Mean ranks for caregiver’s formulation preferences (dispersible tablets: 1.48, SD ±0.71; non-dispersible tablets: 2.12, SD ±0.67; routinely available formulations: 2.40 SD ±0.82) differed significantly (Friedman’s *F* 11.120; *P* < 0.0038); post-hoc testing showed dispersible tablets were preferred over non-dispersible (*P* = 0.018) and routinely available LVX formulations (*P* < 0.001).

**CONCLUSIONS:**

The dispersible LVX 100 mg tablet formulation was preferred and should be prioritised for integration into routine care.

## RÉSUMÉ

**CONTEXTE:**

Nous avons évalué la palatabilité et l'acceptabilité d'un comprimé dispersible de 100 mg et d'un comprimé non dispersible de 250 mg de lévofloxacine (LVX) chez les enfants.

**MÉTHODES:**

*Perform* était un essai randomisé, ouvert et croisé de la biodisponibilité relative des comprimés dispersibles LVX par rapport aux comprimés non dispersibles écrasés chez des enfants âgés de moins de 6 ans recevant régulièrement un traitement préventif contre la TB. Les enfants et les soignants ont rempli des questionnaires de type Likert et de classement sur la tolérance des deux formulations. Nous avons utilisé des statistiques sommaires, comparatives et de classement pour caractériser la tolérance à la formulation.

**RÉSULTATS:**

Au total, 25 enfants ont été recrutés (âge médian : 2,6 ans ; IQR 1,6–4,0). Les soignants ont signalé des problèmes fréquents liés au traitement préventif dans le cadre des soins de routine avant le début de l'étude, notamment le goût des comprimés (*n* = 14, 56%), le fait de vomir ou de recracher les médicaments (*n* = 11, 44%) et le fait que les enfants refusent les médicaments (*n* = 10, 40%). Les soignants ont déclaré que la formulation dispersible était plus facile à prendre pour leur enfant que la formulation non dispersible (*P* = 0,0253). Les classements moyens pour les préférences de formulation des soignants (comprimés dispersibles : 1,48 ; SD ±0,71 ; comprimés non dispersibles : 2,12 ; SD ±0,67 ; formulations couramment disponibles : 2,40 ; SD ±0,82) différaient de manière significative (Friedman's F 11,120 ; *P* < 0,0038) ; les tests post-hoc ont montré que les comprimés dispersibles étaient préférés aux comprimés non dispersibles (*P* = 0,018) et aux formulations LVX couramment disponibles (*P* < 0,001).

**CONCLUSION:**

La formulation dispersible des comprimés de LVX 100 mg a été préférée et devrait être intégrée en priorité dans les soins de routine.

There is a substantial burden of multidrug-resistant TB (MDR-TB; defined as *Mycobacterium tuberculosis* resistant to at least rifampicin and isoniazid) in children, with modelled estimates of 25,000–32,000 cases annually.^1,2^ Additionally, about 2 million children worldwide are estimated to be infected with MDR-TB.^3^ Children <6 years of age are at high risk of progressing to TB disease following infection, and therefore, stand to benefit substantially from MDR-TB preventive treatment. Fluoroquinolones, such as levofloxacin (LVX), are critical for MDR-TB treatment in children.^4–7^ A growing body of research suggests that LVX is safe and well-tolerated in children with minimal adverse events.^8–11^ The possibility of using LVX to prevent TB among drug-resistant exposed children has been explored,^12,13^ and there is a conditional recommendation for its use for this indication by the WHO.^14^

Children and their caregivers’ willingness to use a drug as prescribed determines its overall acceptability.^15^ Formulation palatability is an important characteristic which influences the overall acceptability, treatment adherence and experiences in children and caregivers.^16–18^ Palatability includes a drug’s taste, smell, after-taste and mouthfeel.^19^ Dispersible, taste-masked formulations of LVX used in study settings have demonstrated adequate palatability, with overall high levels of acceptability.^20^ In one study, crushed tablets of LVX reportedly had poor palatability among children being treated for DR-TB.^21^ There are limited data on the palatability of dispersible and other formulations of LVX in children.^20^ In this study, we report on the palatability and acceptability of 100 mg dispersible and crushed non-dispersible 250 mg LVX tablet formulations in children.

## METHODS

### Study design

PERFORM was an open-label randomised 2x2 (two-treatment, two-period) cross-over trial to evaluate the relative bioavailability of dispersible and non-dispersible LVX tablets given according to a weight-banded dosing schedule in children aged <6 years routinely receiving MDR-TB preventive therapy. Briefly, study participants were randomised 1:1 to receive one dose of either the 100 mg dispersible or the 250 mg non-dispersible LVX formulation on the pharmacokinetic (PK) sampling Day 1, followed by the other formulation on the second PK sampling day. The randomisation list was generated by the study statistician using permutated varied blocked randomisation via the *ralloc* procedure in Stata (Stata Corp, College Station, TX, USA). After enrolment, the study pharmacist randomised each study participant using the Sealed Envelope (https://www.sealedenvelope.com/simple-randomiser/v1/ Accessed January 2024) randomisation service. The primary outcomes of the PERFORM trial, including PK and optimised dosing of dispersible and non-dispersible LVX formulations, have been reported elsewhere.^22^

### Setting and study population

In the Western Cape Province, South Africa, a three-drug preventive therapy regimen is used in routine care among children <5 years of age with significant household or other close exposure to an infectious pulmonary MDR-TB source case following exclusion of TB disease through history, clinical examination and plain film chest radiography. If the source case has MDR-TB with susceptibility to the fluoroquinolones, local guidelines recommend LVX (15–20 mg/kg daily), ethambutol (20–25 mg/kg daily) and isoniazid (15–20 mg/kg daily) for 6 months. Children in the PERFORM trial were already receiving a LVX formulation (250 mg non-dispersible tablet, Austell Laboratories, Parktown, South Africa; or 100 mg dispersible tablets; Micro Labs, Bangalore, India) as part of their routine MDR-TB preventive therapy regimen. Children were on at least 2 weeks of MDR-TB preventive therapy, including the routinely used LVX formulation, at the time of enrolment. On PK sampling days, the routinely used formulation of LVX was held and the study formulations administered. The study enrolment period ran from January 2021 to December 2021.

### Eligibility

A participant was eligible for PERFORM if they were age <6 years, taking an MDR-TB preventive therapy regimen that included LVX for at least 14 days prior to enrolment and had provided written informed consent. They were not eligible if they were living with HIV, had any known hypersensitivity or intolerance to LVX, had a serious chronic illness or if they had a Grade 2 or higher laboratory abnormality at the time of screening or within the 14 days prior to enrolment.

### Sampling and recruitment

Children exposed to MDR-TB who were started on routine preventive therapy and evaluated at outpatient paediatric clinics in the Cape Town Metropolitan area, South Africa, were recruited.

### Study formulations and administration

The study medications were LVX 100 mg dispersible tablets and 250 mg non-dispersible tablets (Macleods Pharmaceuticals, Mumbai, India). The study drugs were administered and observed by the study team on the PK days after a fasting period. The dispersible LVX tablets were placed in a dosing cup with 2.5–5.0 mL of water, the cup was swirled to completely disperse the tablet and the liquid was administered. An additional 2.5–5.0 mL of water was added to the cup to suspend any remaining particles and was administered. The non-dispersible tablet was crushed, added to a dosing cup with 2.5–5.0 mL of water, the cup was swirled to completely mix the crushed tablet and the liquid was administered. An additional 2.5–5.0 mL of water was added to the cup and swirled to suspend any remaining particles and was administered. Doses were weight-banded.^9,23^

### Data collection

A standard questionnaire was used to assess the history of use of the routine LVX prior to study entry. Caregivers provided details about how they administered routine LVX at home. They also identified challenges (from a pre-determined list) they had experienced when administering TB preventive treatment. Caregivers responded to 5-point Likert questions asking them how hard/easy it was for their child to take the routine LVX and how their child appeared to feel about the taste and volume of the routine LVX. Children were given the opportunity to provide their assessment of taste of the routine LVX as part of standard of care using a 5-point facial hedonic scale.

The palatability and acceptability assessments of the study dispersible and non-dispersible LVX formulations were then completed on each PK sampling day using a second standard questionnaire. Caregivers responded to 5-point Likert questions asking them how hard/easy it was for their child to take the study LVX, how their child appeared to feel about the taste and volume of the study LVX. Children were given the opportunity to provide their opinion of the taste of the study LVX using a 5-point facial hedonic scale. On the last PK sampling day, the LVX acceptability formulation preferences (routine, dispersible and non-dispersible) were ranked by each study participant’s caregiver. In addition, caregivers were asked to select their preferred LVX formulation in the context of which formulation looked easier to prepare and which formulation they would prefer to give to their child. Children were also given the opportunity to indicate which LVX formulation they preferred (Supplementary Data).

### Statistical analysis

Clinical and demographic characteristics were summarised using descriptive statistics. The analysis was stratified by age into two cohorts (0–<2.0 years vs. 2–<6.0 years), as it was hypothesised that palatability and acceptability of the cohorts would be different since children in these groups are in distinct developmental stages. Palatability and acceptability of routine and study LVX formulations were summarised using frequencies and proportions. Likert data were dichotomised: 1) very easy/easy/not sure vs. hard/very hard, and 2) like very much/like/neutral vs. dislike/dislike very much. The palatability and acceptability for the dispersible and non-dispersible LVX were compared using the McNemar test. The LVX formulation preferences were summarised and ranked using the Friedman test. Post-hoc testing was done using the Dunn-Bonferroni test to adjust for multiple comparisons and the mean ranks, and standard deviations (SDs) were reported along with the corresponding *P*-values. The acceptability data asking the caregivers to select their preferred LVX formulation were summarised using frequencies and proportions and compared using the binomial test. All data were analysed using Stata v16.1 special edition software.

### Ethics statement and details of informed consent

The study was approved by the Health Research Ethics Committee at Stellenbosch University, Tygerberg, South Africa (M20/02/004) and the WHO Ethical Review Committee. Written informed consent was obtained from all caregivers/legal guardians before any trial procedures were performed.

## RESULTS

Twenty-five participants were randomised, enrolled into the study and contributed data on acceptability.^22^ All participants who were enrolled completed the study. The baseline demographic and clinical characteristics are given in Table 1 in aggregate and by age group.

**Table 1.** Baseline demographic and clinical characteristics at enrolment of children on MDR-TB preventative therapy with levofloxacin by age group.

	All participants	Age: 0–<2 years	Age: 2–<6 years
	(*n* = 25)	(*n* = 10)	(*n* = 15)
	*n* (%)	*n* (%)	*n* (%)
Male sex	15 (60.0)	6 (60.0)	9 (60.0)
Race			
Black	8 (32.0)	3 (30.0)	5 (33.3)
Coloured	17 (68.0)	7 (70.0)	10 (66.7)
Age, years, median [IQR]	2.6 [1.6–4.0]	1.4 [0.5–1.6]	3.8 [2.7–4.7]
Weight, kg, median [IQR]	11.9 [10.6–15.0]	10.3 [6.3–10.9]	13.9 [11.9–16.1]
Other TB medications			
Ethambutol	8 (32.0)	2 (20.0)	6 (40.0)
Isoniazid	19 (76.0)	5 (50.0)	14 (93.3)

MDR-TB = multidrug-resistant TB; IQR = interquartile range.

Table 2 shows the acceptability and palatability ratings of LVX provided in routine care. Overall, the most frequent challenges caregivers reported in taking TB preventive medicines were the taste of tablets (14/25, 56%), vomiting/spitting out medicines (11/25, 44%) and children refusing medicines (10/25, 40%). None of the caregivers reported that remembering to give the medicines as a challenge. In total, 21/25 reported it was very easy, easy or felt neutral about taking the routine LVX, while only 13/25 (52%) reported their child seemed to like very much, like or were neutral about the taste of the routine LVX.

**Table 2.** Caregiver and child report of the acceptability and palatability of levofloxacin given in routine care, by age cohort.

	All participants	Age: 0–<2 years	Age: 2–<6 years
	(*n* = 25)	(*n* = 10)	(*n* = 15)
	*n* (%)	*n* (%)	*n* (%)
Routine LVX brand and formulation			
Austell Laboratories,* 250 mg non-dispersible tablet	23 (92)	8 (80)	15 (100)
Micro Labs,^†^ 100 mg dispersible tablets	2 (8)	2 (20)	0 (0)
Relationship to child			
Parent	19 (76)	6 (60)	13 (86.7)
Grandparent	6 (24)	4 (40)	2 (13.3)
Person administering TB preventive medication			
Parent	16 (64)	4 (40)	12 (80)
Grandparent	4 (16)	3 (30)	1 (7)
Parent and grandparent	4 (16)	2 (20)	2 (13)
Both parents	1 (4)	1 (10)	0 (0)
In what form is the routine LVX usually administered?			
Whole tablet (*n* = 10/25)	10 (40)	0 (0.0)	10 (100)
With water	6 (60)	—	6 (60)
With other liquids	0 (0)	—	0 (0)
With food	0 (0)	—	0 (0)
Swallowed whole	2 (20)	—	2 (20)
Nothing added (chewed)	2 (20)	—	2 (20)
Crushed/cut tablet (*n* = 15/25)	15 (60)	10 (67)	5 (33)
With water	13 (87)	8 (80)	5 (100)
With other liquids	1 (7)	1 (10)	0 (0)
With food	1 (7)	1 (10)	0 (0)
Children who have swallowed/taken other medicines before	22 (88)	9 (90)	13 (86.7)
If yes, what types of medicines?			
Tablets	3/22 (14)	0 (0)	3 (23)
Syrups	21/22 (96)	9 (90)	12 (92)
Sprinkles/granules	0/22 (0)	—	—
Which of the following have been challenging when taking TB preventive medicines (‘Yes’ responses)?			
Formulation of medicines	7 (28)	5 (50)	2 (13)
Timing of medicines	2 (8)	2 (20)	0 (0)
Number of tablets	3 (12)	1 (10)	2 (13)
Taste of tablets	14 (56)	9 (90)	5 (33)
Smell of the medicine	1 (4)	1 (10)	0 (0)
Taking the whole dose	5 (20)	3 (30)	2 (13)
Swallowing	9 (36)	7 (70)	2 (13)
Vomiting/spitting up the medicines	11 (44)	6 (60)	5 (33)
Child refusing the medicine	10 (40)	5 (50)	5 (33)
Remembering to give the medicine	0 (0)	0 (0)	0 (0.0)
How easy/hard was it for your child to take routine LVX?			
Very easy, easy or not sure	21 (84)	6 (60)	15 (100)
Hard or very hard	4 (16)	4 (40)	0 (0)
How did the child appear to feel about the taste of the routine LVX?			
Like very much, like or neutral	13 (52)	4 (40)	9 (60)
Dislike or dislike very much	12 (48)	6 (60)	6 (40)
Do you think it would be easy/hard for your child to take study LVX?			
Very easy, easy or neutral	24 (96)	10 (100)	14 (93)
Dislike or dislike very much	1 (4)	0 (0)	1 (7)
When asked, how did the child feel about the taste of the routine LVX?			
Like very much, like or neutral	3 (12)	0 (0)	3 (20)
Dislike or dislike very much	2 (8)	0 (0)	2 (13)
Not applicable (child too young to understand)	20 (80)	10 (100)	10 (67)

* Parktown, South Africa.

^†^ Bangalore, India.

LVX = levofloxacin.

The palatability and acceptability of dispersible and non-dispersible study LVX is displayed in Table 3. Fewer caregivers reported that it appeared hard or very hard for their child to take the dispersible LVX vs. the non-dispersible study LVX (1/25, 4% vs. 6/25, 24%; *P* = 0.025). Regarding taste preferences, respectively 18/25 (72%) and 15/25 (60%) of the caregivers reported their child appeared to like very much, like or were neutral about the taste of the dispersible and non-dispersible levofloxacin; this difference was not statistically significant (*P* = 0.317).

**Table 3.** Palatability and acceptability of dispersible and non-dispersible study LVX by age.

	Dispersible study LVX			Non-dispersible study LVX		
	All participants	Age: 0–<2 years	Age: 2–<6 years	All participants	Age: 0–<2 years	Age: 2–<6 years
	(*n* = 25)	(*n* = 10)	(*n* = 15)	(*n* = 25)	(*n* = 10)	(*n* = 15)
	*n* (%)	*n* (%)	*n* (%)	*n* (%)	*n* (%)	*n* (%)
Relationship to child						
Parent	18 (72)	7 (70)	11 (73)	15 (60)	6 (60)	9 (60)
Grandparent	7 (28)	3 (30)	4 (27)	9 (36)	4 (40)	5 (33)
Sibling	0 (0)	0 (0)	0 (0)	0 (0)	0 (0)	0 (0)
Other	0 (0)	0 (0)	0 (0)	1 (4)	0 (0)	1 (7)
How easy/hard was it for your child to take LVX today?						
Very easy, easy or not sure	24 (96)	9 (90)	15 (100)	19 (76)	8 (80)	11 (73)
Hard or very hard	1 (4)	1 (10)	0 (0)	6 (24)	2 (20)	4 (27)
How did the child appear to feel about the taste of the LVX today?						
Like very much, like or neutral	18 (72)	8 (80)	10 (67)	15 (60)	6 (60)	9 (60)
Dislike or dislike very much	7 (28)	2 (20)	5 (33)	10 (40)	4 (40)	6 (40)
How did the child appear to feel about the volume of the LVX today?						
Like very much, like or neutral	21 (84)	9 (90)	12 (80)	17 (68)	5 (50)	12 (80)
Dislike or dislike very much	4 (16)	1 (10)	3 (20)	8 (32)	5 (50)	3 (20)
When the child was asked, how did they feel about the taste of the LVX today? (*n* = 24)						
Like very much, like or neutral	4 (17)	1 (10)	3 (20)	2 (8)	0 (0)	2 (13)
Dislike or dislike very much	2 (8)	0 (0)	2 (13)	5 (20)	2 (20)	3 (20)
Not applicable, child too young to understand	18 (75)	8 (80)	10 (67)	18 (72)	8 (80)	10 (67)

LVX = levofloxacin.

The Friedman’s test shows there was a statistically significant difference between the overall caregiver LVX preference between the formulations (Q(2) = 11.12, *P* = 0.004). Post-hoc testing indicated the mean ranks were statistically significant between the 100 mg dispersible and 250 mg non-dispersible study LVX (1.48, SD ±0.71 vs. 2.12, SD ±0.67, respectively; *P* = 0.018) and the 100 mg dispersible and routine drug formulations (1.48, SD ±0.71 vs. 2.40, SD ±0.82, respectively; *P* < 0.001). There was no difference between the 250 mg non-dispersible and routine drug formulations (2.12, SD ±0.67 vs. 2.40, SD ±0.82, respectively; *P* = 0.685). Overall, 18/25 (72.0%) caregivers thought the dispersible study LVX seemed easier to prepare in comparison to the non-dispersible and routine study LVX (3/25, 12.0% and 4/25, 16.0%, respectively; *P* < 0.001). Similarly, 17/24 (70.8%) caregivers reported preferring to give their child the dispersible study LVX tablet in comparison to those who preferred the non-dispersible LVX formulation and routine LVX formulations (3/24, 12.5% and 4/24, 16.7%, respectively; *P* < 0.001).

## DISCUSSION

In this trial, using an efficient cross-over design, we demonstrate that a commercially available dispersible tablet formulation of LVX was preferred to non-dispersible tablets among young children receiving MDR-TB preventive therapy, and also among their caregivers.

Our study found that caregivers of children reported frequent issues with medication taste, vomiting and treatment refusal using the routinely available formulations of LVX prior to study entry. This highlights how common these challenges are for caregivers and the need for more acceptable formulations. Although we did not assess adherence in our trial, previous studies have shown that lack of medication acceptability is an important contributor to poor adherence.^24^

In the direct comparisons between study formulations, caregivers reported the dispersible study LVX tablet as being easier for their child to take in comparison to the non-dispersible study LVX formulation. There was not a significant difference in taste between the two study formulations. Overall, caregivers preferred the dispersible tablet formulation, and said that it appeared easier to prepare and administer compared to both the 250 mg non-dispersible study and routine formulations. We found no differences between the routine and 250 mg formulations, in terms of perceived taste and ease of preparation and administration. These data are consistent with a previous study reporting that the same dispersible formulation of LVX used in our current study had good palatability and was preferred by caregivers.^20^ However, this previous study was limited by its design, as it did not directly evaluate both formulations in the same child. Instead, the study relied on caregiver recall of the acceptability of the non-dispersible tablet that was given in combination with other drugs in the standard-of-care regimen. This further diluted participants’ ability to discern the acceptability of one drug among the others. Our study used a more robust direct comparison method of the two formulations, and confirms and strengthens the previous report of the overall preference for the dispersible formulation. The majority of caregivers in our study reported that the volume of treatment administered was acceptable, an important consideration, especially for young children.^25^

Palatability has been reported as one of the most important characteristics of medication acceptability among young children and their caregivers.^26,27^ This has been confirmed specifically for TB preventive treatment.^27^ Bitter tasting medication can cause nausea and vomiting, potentially affecting drug absorption and health outcomes.^28,29^ Our findings showing improved ease of administration and overall preference for the dispersible tablet formulations have important practical implications. The dispersible LVX tablet studied here is commercially available, including through the Global Drug Facility, and it and similar dispersible formulations should be provided as a priority for children needing LVX for rifampicin-resistant TB treatment. LVX is also recommended by the WHO as an option for TB prevention in high-risk children exposed to MDR-TB. Two phase III clinical trials evaluating whether LVX is safe and efficacious for preventing MDR-TB in adults and children are expected to provide results in late 2023.^30,31^ Should these trials provide support for this indication, LVX uptake in children worldwide would be expected to increase substantially. Palatable formulations will be critical for supporting LVX preventive treatment in children and should be prioritised. The 100 mg dispersible formulation also offers dosing flexibility over non-dispersible 250 mg formulations, important especially for the smallest children.^20,32^

One limitation of this study is that children’s preferences were based on caregivers’ perceptions of their reactions to the different treatment formulations. However, given that the majority of children were younger than six, methods of assessment other than careful observation by caregivers are limited. The small sample size of the study population may have limited our ability to detect differences between formulation, including those related to taste. Additionally, the administration setting of the study formulations, which occurred during intensive PK blood sampling, was different to the administration setting of routine LVX, which was less restrictive and in their home or other desired location without restrictions. In future research endeavours, it is advisable to assess the acceptability and palatability of drug formulations not solely during intensive PK samplings, but rather in households or comparable settings. This approach would mirror conditions more akin to routine medication administration, outside the potentially stressful confines of a study environment. Another weakness is the limited aspects of treatment palatability that were assessed, including possible changes to perceptions about the palatability over time. In future research, additional aspects of treatment palatability, such as smell, mouth-feel and after-taste could be assessed if children older than those in this cohort are included.

Our findings demonstrate that dispersible anti-TB formulations should be prioritised for development among children and their caregivers. Further research that includes children earlier on in the drug development stages is needed to improve formulation palatability. Effective and safe medicines that are also acceptable and palatable are needed to optimally serve our youngest and most vulnerable populations in the fight against TB. Furthermore, longitudinal research among children and their caregivers in routine care is essential to better understand the context in which treatment is administered, and its impact on overall treatment acceptability, adherence and outcome.

## Supplementary Material


