# Supplementary material for: Acceptability of levofloxacin dispersible and non-dispersible tablet formulations in children receiving TB preventive treatment
Source: IJTLD Open. 2024 Feb 1;1(2):69–75. doi: 10.5588/ijtldopen.23.0462 (PMC11221592; doi:10.5588/ijtldopen.23.0462)
Supplement: Supplementary file 1 [file iutld_ijtld_open_23.0462_supplementarydata1.pdf]

PERFORM Trial - Levofloxacin Acceptability History Form

**Note: This form relates to the routine medication that the child is currently taking, NOT the study medication**

1. Who administered this questionnaire? ☐ **Nurse** ☐ **Doctor** ☐ **Counsellor** ☐ **Other** *(specify)*____________________

2. Which levofloxacin brand is child taking in routine care? (*tick one*)

☐ Austell laboratories

☐ Macleod's Pharmaceuticals

☐ Other (*Specify*)____

3. What is your relationship to the child? *(tick one)*

☐ **Mother** ☐ **Father** ☐ **Brother** ☐ **Sister** ☐ **Cousin**

☐ **Grandmother** ☐ **Grandfather** ☐ **Uncle** ☐ **Aunt** ☐ **Friend**

☐ **Other** *(Specify)* ____

4. Who has been giving TB preventive medication to the child? *(tick one)*

☐ **Mother** ☐ **Father** ☐ **Brother** ☐ **Sister** ☐ **Cousin**

☐ **Grandmother** ☐ **Grandfather** ☐ **Uncle** ☐ **Aunt** ☐ **Friend**

☐ **Other** *(Specify)* ____

5. In what form do you usually administer ***routine*** levofloxacin to the child at home? *(tick one)*

**Whole tablet:**	☐ With water	☐ With other liquid, specify________________	☐ With food	☐ Nothing added (swallowed whole)	☐ Nothing added (Chewed)
**Crushed / Cut tablet:**	☐ With water	☐ With other liquid, Specify________________	☐ With food	☐ Nothing added **(swallowed as is)**	☐ Nothing added (Chewed)

6. Has the child ever swallowed/taken any other medicines before? ☐ **Yes** ☐ **No**
*(if **No** go to Q.7)*

a) If **Yes** which types of medicines has the child swallowed/taken before? *(tick all that apply)*

☐ **Tablets** ☐ **Syrups** ☐ **Sprinkles/Granules**

b) Any comments:

____

There are many reasons why people do not like to take their medicines. We know that is difficult taking medicines every day. Most people miss doses from time to time and it is rare that people take medication perfectly. We are interested in finding out what it is like for you and your child

7. A. Do you think any of the following have been challenging for you or for the child taking TB preventive medicines? *(tick all that apply)*

	Yes	No		Yes	No
**i.** Formulation of medicines (ie only tablet available, no liquid/dissolvable tablets):	☐	☐	**vi.** Taking the whole dose:	☐	☐
**ii.** Timing of medicines:	☐	☐	**vii.** Swallowing:	☐	☐
**iii.** Number of tablets:	☐	☐	**viii.** Vomiting/spitting up the medicines:	☐	☐
**iv.** Taste of tablets:	☐	☐	**ix.** Child refusing the medicine:	☐	☐
**v.** Smell of the medicine:	☐	☐	**x.** Remembering to give the medicine:	☐	☐

B. Comments:

____

____

____

____

8. How easy/hard has it been for your child to take routine ***levofloxacin*** medicine? *(tick one)*

☐ **Very easy** ☐ **Easy** ☐ **Not sure** ☐ **Hard** ☐ **Very hard**

9. In your assessment, how does the child appear to feel about the taste of the routine ***levofloxacin*** medicine? (*tick one)*

☐ **Dislike very much** ☐ **Dislike** ☐ **Neutral** ☐ **Like** ☐ **Like very much**

10. Do you think it will be easy/hard for your child to take ***study levofloxacin*** *medicine? (tick one)*

☐ **Very easy** ☐ **Easy** ☐ **Not sure** ☐ **Hard** ☐ **Very hard**

**11. Question for the child:** How did you feel about the taste of your ***levofloxacin*** medicine? (show the child the medicine)				
11.1. Dislike very much	11.2. Dislike	11.3. Neutral	11.4. Like	11.5. Like very much

☐ Not applicable (child too young to understand)

PERFORM STUDY
PK Visit Acceptability Form

1. Who administered this questionnaire? ☐ **Nurse** ☐ **Doctor** ☐ **Counsellor** ☐ **Other** *(specify)*_____________________

2. Which ***study levofloxacin (Macleods)*** was administered today? *(tick one)*

☐ **100 mg dispersible with water** ☐ **250 mg non-dispersible crushed with water**

3. To the responder: What is your relationship to the child? *(tick one)*

☐ **Mother** ☐ **Father** ☐ **Brother** ☐ **Sister** ☐ **Cousin**
☐ **Grandmother** ☐ **Grandfather** ☐ **Uncle** ☐ **Aunt** ☐ **Friend**
☐ **Other** *(Specify)* ____

4. How easy/hard was it for your child to take ***study levofloxacin*** medicine today? *(tick one)*

☐ **Very easy** ☐ **Easy** ☐ **Not sure** ☐ **Hard** ☐ **Very hard**

5. In your assessment, how did the child appear to feel about the taste of the ***study levofloxacin*** medicine today? (*tick one)*

☐ **Dislike very much** ☐ **Dislike** ☐ **Neutral** ☐ **Like** ☐ **Like very much**

6. In your assessment, how did the child appear to feel about the amount (volume) of the ***study levofloxacin*** medicine today? (*tick one)*

☐ **Dislike very much** ☐ **Dislike** ☐ **Neutral** ☐ **Like** ☐ **Like very much**

Ask the patient (child) to indicate their answer within 15 minutes of taking the ***study levofloxacin*** *dose*

**7.** How did you feel about the taste of the ***study levofloxacin*** medicine today? (patient)				
7.1. Dislike very much	7.2. Dislike	7.3. Neutral	7.4. Like	7.5. Like very much

☐ Not applicable (child too young to understand)

Is this the last PK day? ☐ **Yes** ☐ **No, skip**

8. Overall, which type of the levofloxacin medicine **do you think** your child prefers to take? Enter one number for each formulation *(1 -First preference (best), 2-Second preference, 3-Third preference (worst))*

☐ **100 mg dispersible with water** ☐ **250 mg tablet crushed with water** ☐ **routinely available tablet with water**

9. Overall, which type of the levofloxacin medicine **do you think** looks easier to prepare? *(tick one)*

☐ **100 mg dispersible with water** ☐ **250 mg tablet crushed with water** ☐ **routinely available tablet with water**

10. Overall, which type of the levofloxacin medicine would you prefer to give to your child? *(tick one)*

☐ **100 mg dispersible with water** ☐ **250 mg tablet crushed with water** ☐ **routinely available tablet with water**

11. If appropriate for the child's age and understanding please get **the child** to indicate which levofloxacin medicine they think they prefer: *(tick one)*

☐ **100 mg dispersible with water** ☐ **250 mg tablet crushed with water** ☐ **routinely available tablet with water**

☐ Not applicable (child too young to understand)

12. Any additional comments from parent/caregiver?

____

____
